# The effectiveness of active surveillance measures for COVID‐19 cases in Pudong New Area Shanghai, China, 2020

**DOI:** 10.1002/jmv.26805

**Published:** 2021-02-16

**Authors:** Hanzhao Liu, Chuchu Ye, Yuanping Wang, Weiping Zhu, Yifeng Shen, Caoyi Xue, Hong Zhang, Yanyan Zhang, Shihong Li, Bing Zhao, Hongmei Xu, Lipeng Hao, Yixin Zhou

**Affiliations:** ^1^ Research Base of Key Laboratory of Surveillance and Early Warning of Infectious Disease of China CDC Pudong New Area Center for Disease Control and Prevention Shanghai China; ^2^ Pudong Institute of Preventive Medicine Fudan University Shanghai China

**Keywords:** close contacts, COVID‐19, potential exposure populations, quarantine, surveillance

## Abstract

The aim of this study was to thoroughly document the effects of multiple intervention and control methods to mitigate the ongoing coronavirus disease 2019 (COVID‐19) outbreak in Pudong New Area, Shanghai. After identification of the first confirmed case of COVID‐19 in Pudong on January 21, 2020, the local Center for Disease Control and Prevention (CDC) launched a case investigation involving isolation, close‐contact (CC) tracing and quarantine of persons with a potential exposure risk to prevent and control transmission. Epidemiological features of cases detected by three different strategies were compared to assess the impact of these active surveillance measures. As of February 16, 2020, a total of 108 confirmed COVID‐19 cases had been identified in Pudong, Shanghai. Forty‐five (41.67%) cases were identified through active surveillance measures, with 22 (20.37%) identified by CC tracing and 23 (21.30%) by quarantine of potential exposure populations (PEPs). The average interval from illness onset to the first medical visit was 1 day. Cases identified by CC tracing and PEPs were quarantined for 0.5 and 1 day before illness onset, respectively. The time intervals from illness onset to the first medical visit and isolation among actively screened cases were 2 days (*p* = .02) and 3 days (*p* = .00) shorter, respectively, than those among self‐admission cases. Our study highlights the importance of active surveillance for potential COVID‐19 cases, as demonstrated by shortened time intervals from illness onset to both the first medical visit and isolation. These measures contributed to the effective control of the COVID‐19 outbreak in Pudong, Shanghai.

## Research Highlights

‐ Nearly half of the confirmed COVID‐19 cases in Pudong New Area, Shanghai were detected through those active surveillance measures in early stage of the pandemic.

‐ Cases identified from CCs and PEPs were quarantined for 0.5 and 1 day before illness onset, respectively.

‐ The time intervals from illness onset to the first medical visit and isolation among actively screened cases were 2 and 3 days shorter respectively than those among self‐admission cases.

‐ Early tracing and quarantine of specific populations with a higher exposure risk may have a positive effect on pandemic control in the community.

## 1 INTRODUCTION

As of June 10, 2020, coronavirus disease 2019 (COVID‐19), caused by severe acute respiratory syndrome coronavirus 2 (SARS‐CoV‐2), has infected more than 31.05 million individuals and caused more than 962,482 deaths globally.^1^ Increasing evidence indicates that COVID‐19 is transmitted by respiratory droplets from coughing and sneezing. People are generally susceptible, and the incubation period ranges from 2 to 14 days.^2^ As a recently emerged novel coronavirus, SARS‐CoV‐2 causes more severe illness than seasonal influenza viruses, with an estimated reproductive number of approximately 2.2–3.6,^3^, ^4^, ^5^ which is nearly twice as high as that of seasonal influenza viruses.^6^

Containment and suppression are the two major strategies conducted in China.^7^ Since late January 2020, measures including rapid identification and isolation of cases, active monitoring and quarantine of CCs, and border controls have been implemented to reduce transmission, thereby delaying the timing and reducing the size of the epidemic peak in China.^8^ These containment measures have been demonstrated to play a substantial role in determining whether an outbreak is controllable by consistent results from many model studies.^9^, ^10^ Modeling studies have estimated that, without these containment efforts, the number of COVID‐19 cases would have been approximately 67‐fold higher than the current number to date.^11^ Despite the increase in articles related to prevention and control, more field epidemiological evidence is needed.^12^

Shanghai, as a large city in eastern China with a population of approximately 33 million, has faced a significant risk of the COVID‐19 outbreak since the start of 2020. Apart from routine surveillance and management of CCs, in Pudong New Area, the biggest district in Shanghai city, we have also conducted active identification and quarantine for potential exposure populations (PEPs). A PEP refers to residents or travelers from areas with a higher infection risk than Shanghai. In this study, we compared differences in demographics among cases identified through symptom‐based surveillance of persons who sought medical care on their own, monitoring of both CCs and PEPs, and estimated serial intervals, such as those from illness onset to quarantine, the first medical visit, isolation, and confirmation, to assess the impact of these active surveillance measures.

## 2 MATERIALS AND METHODS

### 2.1 Active surveillance and quarantine of PEPs

On January 21, 2020, the first COVID‐19 case was identified in Pudong New Area, Shanghai. The patient had a travel history to Wuhan, Hubei Province, China. Three days later, screening of travelers from Hubei Province for symptoms of COVID‐19 began in all communities and at every city entrance in Pudong. Visitors or residents who arrived in Pudong on or after January 12, 2020, from Hubei Province were defined as PEPs. Community screening was conducted to identify PEPs who had already arrived in the Pudong New Area. Moreover, any new arrivals from Hubei Province were screened at every city entrance, including airports, train stations, and so on. All the information of the identified PEPs at city entrances was immediately sent by a big data network to the community in which they lived. PEPs were quarantined under compulsory medical observation at home or centralized facilities for 14 days, and during that time, they were assessed for fever or respiratory symptoms by medical staff twice daily. Persons in quarantine were transferred by ambulance directly to fever clinics when they developed any related symptoms during the quarantine period, while others were released after 14 days without fever and any respiratory symptoms.

### 2.2 COVID‐19 case detection and management

Patients transferred by ambulance from centralized facilities or their homes and people with clinical manifestations of COVID‐19 who sought medical care on their own were sent to fever clinics immediately when they visited a hospital. According to the COVID‐19 case definition in Shanghai,^13^ suspected cases were diagnosed and sampled in fever clinics. Within 2 h, the information of suspected cases was reported online, and specimens were sent to the Pudong Center for Disease Control and Prevention (CDC) for testing by real‐time reverse transcription‐polymerase chain reaction (RT‐PCR). Moreover, patients were isolated in the hospital until COVID‐19 confirmation and then transferred to a designated hospital for isolation and treatment. The other suspected cases were excluded by two consecutive negative laboratory test results of samples taken at intervals of more than 24 h.

### 2.3 Identification and quarantine of CCs

An epidemiological investigation of all suspected cases was conducted by the Pudong CDC within 24 h to identify CCs and collect basic demographics, signs, symptoms, and exposure histories. CCs were identified through contact tracing and defined as those who lived in the same apartment, shared a meal, traveled, or socially interacted, and had close (within 1 m) and prolonged (generally ≥15 min) contact with any suspected COVID‐19 patients without effective protection from 2 days before the patient's illness onset to the time of patient isolation. Detailed information of CCs was sent to their communities for further confirmation and management. Similar to PEPs, the CCs were quarantined, but only at centralized facilities for 14 days. The release was conditional on a negative RT‐PCR result for the related suspected case or the absence of fever and any respiratory symptoms for 14 days if the related suspected case was confirmed.

**Figure 1:**
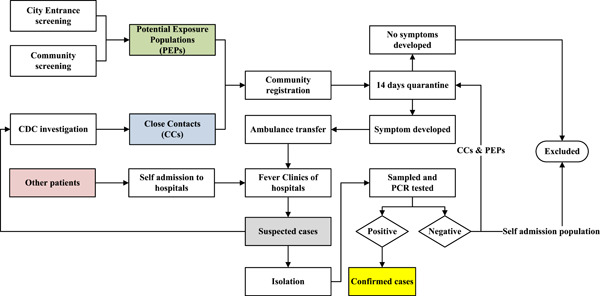
Flowchart of active surveillance for COVID‐19 cases in Pudong New Area, Shanghai, January–February 2020

Figure 1 shows a flowchart of active surveillance for COVID‐19 cases in Pudong New Area, Shanghai, January–February 2020.

### 2.4 Data analysis

All confirmed COVID‐19 cases reported between January 21 and February 16, 2020, were investigated by the Pudong CDC. Analyses included the following: (1) summarizing case characteristics, (2) determining the age distribution and sex ratio, and (3) constructing an epidemiological curve. Specifically, we compared the epidemiological features of patients identified by three different detection methods: CC management, PEP management, and screening of self‐admitted patients in fever clinics. Important intervention dates were plotted to compare intervals from illness onset to quarantine initiation, the first medical visit, isolation, and confirmation.

All statistical analyses were performed using R 3.5.1 (R Core Team, R: A language and environment for statistical computing. R Foundation for Statistical Computing, Vienna, Austria). The *χ* ^2^ test or Fisher's exact test was used for categorical variables, and the Wilcoxon rank sum test or Kruskal–Wallis test was used for continuous variables, as appropriate. *p* < .05 were considered statistically significant.

## 3 RESULTS

Between January 21 and February 16, 2020, the Pudong CDC confirmed 108 COVID‐19 cases (Table 1). The median age was 47 years (interquartile range = 36.75–64 years), and 60 (55.56%) patients were male. The proportion of older adults aged 60 years and above (*n* = 37, 34.26%) was the highest among four age groups, followed by adults aged 40–59 years (*n* = 36, 33.33%) and younger adults aged 20–39 years (*n* = 32, 29.63%). The youngest cases involved three teenagers aged 18 years, accounting for 2.78% of all cases.

**Table 1:** Characteristics of the confirmed COVID‐19 patients in Pudong New Area, Shanghai, January–February 2020

	Case detection method, *N* (%)			
Characteristics	Overall	Self‐admission to fever clinic	Close contact tracing	Active monitoring of potential exposure populations
Case no.	108 (100)	63 (58.33)	22 (20.37)	23 (21.30)
Sex of cases				
Female	48 (44.44)	25 (39.68)	14 (63.64)	9 (39.13)
Male	60 (55.56)	38 (60.32)	8 (36.36)	14 (60.87)
Age (years), median (IQR)	47 (36.75 to 64)	47 (38 to 64)	52.5 (35.25 to 67.75)	42 (36.5 to 58)
Age groups (years)				
0–19	3 (2.78)	2 (3.17)	1 (4.55)	0 (0.00)
20–39	32 (29.63)	18 (28.57)	7 (31.82)	7 (30.43)
40–59	36 (33.33)	22 (34.92)	4 (18.18)	10 (43.48)
60–	37 (34.26)	21 (33.33)	10 (45.45)	6 (26.09)
Epidemiological link to Hubei Province				
Exposure to persons with travel history to Hubei Province	16 (14.81)	10 (15.87)	6 (27.27)	0 (0.00)
Travel history to Hubei Province	69 (63.89)	34 (53.97)	12 (54.55)	23 (100.00)
Without Hubei‐related exposure	23 (21.30)	19 (30.16)	4 (18.18)	0 (0.00)
Interval of illness onset to the first medical visit, median (IQR)	1 (0 to 4)	2 (0.5 to 4.5)	1 (0‐3.75)	0 (0 to 1.5)
Interval of illness onset to quarantine, median (IQR)	−1 (−3 to 1)	–	−0.5 (−3.5 to 1)	−1 (−3 to 0.5)
Interval of illness onset to isolation, median (IQR)	2 (1 to 6)	4 (2 to 8)	1.5 (0 to 3.75)	1 (0 to 2)
Interval of illness onset to confirmation, median (IQR)	4 (2 to 7.25)	6 (3 to 9)	2.5 (1 to 5.75)	2 (1 to 4.5)

Abbreviations: COVID‐19, coronavirus disease 2019; IQR, interquartile range.

According to the epidemiological investigation, a total of 69 (63.89%) cases had a travel history to Hubei province, and 85.51% (59/69) of these patients lived in or traveled to Wuhan city. Twenty‐three (33.33%) of these 69 cases were identified through active monitoring of PEPs, 12 (17.39%) were identified from CCs, and 34 (49.28%) were self‐admitted patients identified by fever clinics at local hospitals. In addition, 16 (14.81%) cases without a Hubei travel history had been exposed to persons who traveled to Hubei before contact. The other 23 cases (21.30%) had no epidemiological link with Hubei Province (Table 1).

Fever clinics reported 63 COVID‐19 cases in Pudong New Area, accounting for 58.33% of all cases. Twenty‐two (20.37%) cases were identified from CC tracing, and 23 (21.30%) were detected from active monitoring of PEPs.

**Figure 2:**
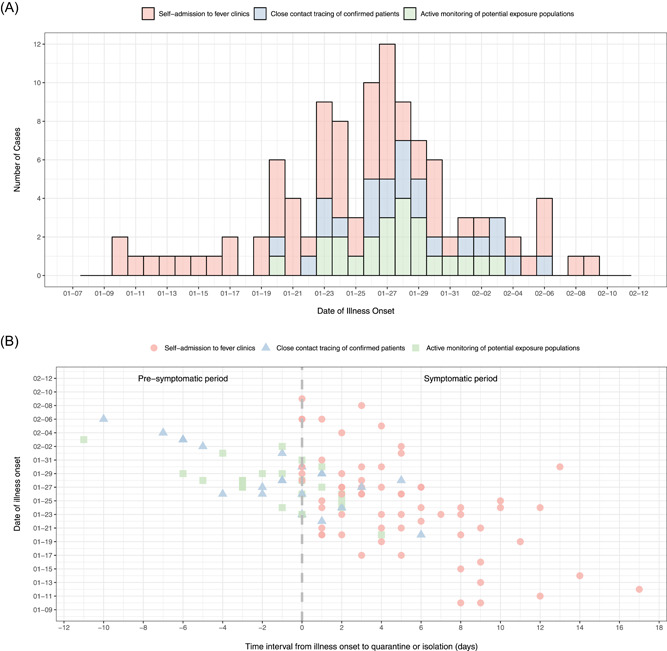
The epidemic curve and time interval from illness onset to isolation/quarantine of confirmed COVID‐19 cases with different detection methods in Pudong New Area, Shanghai, January–February 2020. (A) The epidemic curve of confirmed COVID‐19 cases in Pudong New Area, Shanghai, China, January–February 2020. (B) The time interval from illness onset to quarantine/isolation of 108 confirmed COVID‐19 cases in Pudong New Area, Shanghai, China, January–February 2020. COVID‐19, coronavirus disease 2019

Within 2 days of illness onset, 71 (65.74%) patients visited the hospital, 55 (50.93%) were isolated by the hospital, and 34 (31.48%) were confirmed. The number of reported confirmed cases increased sharply from January 23, when Wuhan initiated lockdown measures,^14^ and peaked approximately 1 week later. The epidemic was effectively controlled within 1 month, and no additional cases were identified at the end of February (Figure 2A).

On average, quarantine was initiated 1 day before illness onset among those with a potential exposure risk and 0.5 days before illness onset among CCs. A total of 52.17% (12/23) of the PEPs and 50.00% (11/22) of the CCs were quarantined before illness onset (Figure 2B).

**Figure 3:**
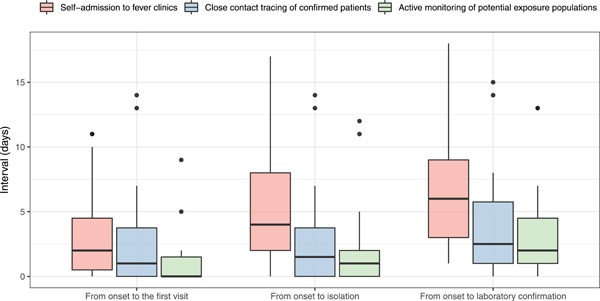
Time intervals from illness onset to the first visit, isolation, and confirmation of confirmed COVID‐19 cases with different detection methods in Pudong New Area, Shanghai, January–February 2020

The median intervals from illness onset to the first visit, isolation, and confirmation were 1, 2 and 4 days, respectively. Compared to those of self‐admission cases, the intervals from illness onset to the first medical visit, isolation, and confirmation of cases identified from CCs and PEPs were all significantly shorter (*p* = .02, .00, and .00, respectively; Table 1 and Figure 3).

## 4 DISCUSSION

Nearly half of the confirmed COVID‐19 cases in the Pudong New Area were detected through active surveillance measures, according to our study. Large proportions of the CCs and potentially exposed individuals were identified and quarantined before the development of symptoms. On average, quarantine was initiated 1 day before the onset of illness among potentially exposed individuals and 0.5 days before the onset of illness among CCs. The time interval from illness onset to isolation for patients identified by active surveillance was significantly shorter than that for self‐admitted patients.

At the beginning of the intervention, all of our surveillance measures of monitoring the population with higher infected risk, such as close contacts and travelers from high‐risk areas, aimed to identify and isolate them as soon as they became patients. We did not actually consider avoiding presymptomatic transmission at that time. However, with the implementation of the intervention measures, increasing evidence from related research globally had indicated the occurrence of asymptomatic and presymptomatic transmission of COVID‐19,^15^, ^16^ which increase the necessity of monitoring population with high infectious risk before they became a patient. Our results also showed that a lot of cases had been quarantined before they were detected with SARS‐CoV‐2.

As the prodromal symptoms are mild and do not begin abruptly, early signs and symptoms of COVID‐19 are difficult to recognize.^17^, ^18^ Given the individual differences in the symptoms of this disease as well as subjective and constantly changing symptoms, most patients might not seek prompt medical care when they develop any symptom, especially at the early stage of a pandemic. A lack of timely detection of cases directly leads to an increased risk of community transmission; however, these problems can be averted by active quarantine and compulsory medical observation of the specific population. At the early stage of the COVID‐19 outbreak, the proportion of persons who visited a fever clinic within 2 days of illness onset was 65.74% in Pudong New Area, Shanghai, compared with 61% in Gansu Province of China,^19^ 27% in Wuhan in China,^20^ and 23.1% in South Korea.^21^ Patients with mild symptoms promptly sought timely medical care, and detection and isolation were performed within a short period, which significantly reduced CC transmission, the risk of community transmission, and the likelihood of a patient developing severe illness. Overall, 74.07% of the patients in our study were isolated within 5 days after illness onset, which is similar to the rate of 68% in Gansu^19^ and higher than the rate of 11% in Wuhan at the early stages of the disease.^7^ In Singapore, the mean interval from symptom onset to hospital isolation or quarantine was 5.6 days.^22^ Routine health management ensured timely case identification and confirmation, effectively protecting additional community members from infection. As a benefit from the positive control measures, local transmission was effectively controlled after 1 month from January 21 in Pudong New Area, Shanghai.

We are currently facing a totally new, extraordinarily complex and highly damaging virus. Active surveillance of cases and management, coupled with identification and quarantine of PEPs and CCs, is an effective strategy and is significantly more effective for preventing infection than travel restrictions and exposure restrictions.^11^ These active surveillance measures have been successfully implemented in several countries, including Singapore and South Korea. Singapore maximized detection of suspected patients and legally supported home quarantine orders for patients with mild illness.^23^ South Korea substantially expanded the scope of testing to detect and isolate cases as early as possible.^24^

This investigation has two limitations. First, we did not calculate the actual number of cases averted by each of our strategies due to a lack of information from the limited cases. However, previous studies using epidemiological models have demonstrated that early identification of potential cases plays a substantial role in determining whether an outbreak is controllable.^10^, ^25^, ^26^ Second, the intervention we conducted in Pudong might need strong policy support from the government and need effective cooperation among the airport, CDC, hospitals, community health service centers, police stations, and so forth. It may not be easily implemented in other countries.^27^ However, we believe that the principle to control potentially infected population (including close contacts and population from the high‐risk area) could be considered for COVID‐19 control globally, and the successful practice in Pudong would bring insights into a reference.

## 5 CONCLUSIONS

Our study indicated that active surveillance of the target population plays a critical role in identifying COVID‐19 cases, especially during the early stage of an epidemic.

## CONFLICT OF INTERESTS

The authors declare that there are no conflict of interests.

## PEER REVIEW

The peer review history for this article is available at https://publons.com/publon/10.1002/jmv.26805.

## AUTHOR CONTRIBUTIONS

Yixin Zhou, Lipeng Hao, and Weiping Zhu designed the study. Hanzhao Liu, Chuchu Ye, Yuanping Wang, and Hongmei Xu conducted the surveillance and analyzed the data. Yifeng Shen, Caoyi Xue, Hong Zhang, and Shihong Li collected and cleaned the surveillance data. Yanyan Zhang and Bing Zhao conducted the PCR test for all the samples. Hanzhao Liu and Chuchu Ye wrote the manuscript.

## Data Availability

The data that support the findings of this study are available from the corresponding author upon reasonable request.
